# Using Friendship Ties to Understand the Prevalence of, and Factors Associated With, Intimate Partner Violence Among Adolescents and Young Adults in Kenya: Cross-Sectional, Respondent-Driven Survey Study

**DOI:** 10.2196/19023

**Published:** 2020-12-31

**Authors:** Peter Memiah, Anne Kamau, Yvonne Opanga, Samuel Muhula, Emmanuel Nyakeriga, Felix Humwa, Courtney Cook, Caroline Kingori, Job Muriithi

**Affiliations:** 1 University of Maryland School of Medicine Baltimore, MD United States; 2 University of Nairobi Nairobi Kenya; 3 Amref Health in Kenya Nairobi Kenya; 4 University of California, San Francisco Nairobi Kenya; 5 Fortis Institute Pensacola, FL United States; 6 Ohio University Athens, OH United States

**Keywords:** intimate partner violence, adolescents, young adults, bullying, physical abuse, abuse, Africa, prevalence, risk

## Abstract

**Background:**

Optimization of innovative approaches is required for estimating the intimate partner violence (IPV) burden among adolescents and young adults (AYA). Further investigation is required to identify risk and protective factors associated with IPV among AYA. There remain significant gaps in understanding these factors among this vulnerable population.

**Objective:**

The goal of our study was to determine the prevalence of IPV among an urban population of AYA and to identify factors associated with IPV among AYA.

**Methods:**

A cross-sectional study design utilizing respondent-driven sampling was adopted. The study was conducted among 887 AYA, aged 15 to 24 years, residing in Nairobi, Kenya. Data were collected through a phone-based survey using the REACH (Reaching, Engaging Adolescents and Young Adults for Care Continuum in Health)-AYA app. Questions on behavioral and psychosocial factors were adopted from different standardized questionnaires. Descriptive, bivariate, and multivariable statistics were used to describe the characteristics of the study sample.

**Results:**

Of the 887 participants, a higher proportion were male (540/887, 60.9%) compared to female (347/887, 39.1%). The prevalence of IPV was 22.3% (124/556). IPV was associated with being unsure if it was okay for a boy to hit his girlfriend, living in a home with physical violence or abuse, and being bullied (*P*=.005). The likelihood of experiencing IPV was higher among respondents whose friends and family members used alcohol (odds ratio [OR] 1.80, 95% CI 1.09-2.98) and among those who had repeated a class at school in the past two years (OR 1.90, 95% CI 1.11-3.23). Respondents who visited a health facility or doctor for reproductive health services were 2 times more likely to experience IPV (OR 2.23, 95% CI 1.40-3.70). Respondents who had used illicit drugs were 2 times more likely to experience IPV (OR 4.31, 95% CI 2.64-7.04). The probability of experiencing IPV decreased by 63% (OR 0.37, 95% CI 0.16-0.85) among respondents who refused to have sex with someone who was not prepared to use a condom.

**Conclusions:**

IPV remains a significant public health priority because of its impact to society. Our results are in congruence with other similar studies. Efforts toward incorporating appropriate IPV core measures into the comprehensive care package for every AYA seeking health services should be explored. Programs need to address constellations of risk and protective factors linked to IPV in an effort to prevent its occurrence.

## Introduction

While efforts have been made in Kenya to address gaps in intimate partner violence (IPV) programming [1], little has been done to understand the epidemic from the perspective of adolescents and young adults (AYA) [2]. The World Health Organization (WHO) reports a 19% to 66% lifetime prevalence of IPV among AYA who are between the ages of 10 and 24 years [3]. IPV is characterized as behavior by a partner that causes emotional, sexual, or physical abuse, as well as other controlling behaviors, and generally occurs from adolescence to adulthood [4,5]. Consequently, IPV survivors are at risk of suffering from poor social and health outcomes related to reproductive health, substance use, sexual health, and mental health [6].

Risk factors for AYA operate at multiple levels that include individual, dyadic (ie, interactions with peers, partners, or parents), community (ie, school environment), and societal levels [7]. At the individual level, young age, low level of education, childhood sexual abuse, drug and alcohol use, and mental health are some of the risk factors for IPV. Economic hardships, relationship conflicts, and patriarchy have been associated with IPV risk at the dyadic level. Furthermore, lack of legal sanctions against IPV and women’s civil rights, violence, poverty, and gender inequality norms are associated with community- and societal-level factors [8]. Thus, the identification of such multifaceted determinants of risky behaviors is critical in informing contextually relevant interventions in Kenya [9,10]. Risk factors can be mitigated by employing protective factors, such as social support, quality friendships, access to resources, funding for services to support community-based initiatives and interventions, and community cohesiveness [7].

In Kenya, there are significant gaps in IPV-prevention knowledge among AYA. Prevention strategies should be developed with nuance, especially because there is a lack of adequate well-coordinated efforts incorporating interventions that address biological, behavioral, psychosocial, and structural factors among AYA in Kenya [11]. Adolescents are avid users of mobile devices, and frequent virtual communication among adolescents has been shown to strengthen the quality of existing relationships [12]. Network analysis and content analysis of adolescent online communication shows that most of their online communication involves positive interactions between friends, and that mobile devices are used as tools for better understanding and supporting their positive development [13]. Access to mobile phones among young people is steadily increasing, with no difference in penetration between formal and informal settings [14]. The rapid increase in mobile penetration in Kenya provides platforms for social interactions and engagements [15], with AYA more likely to own smartphones. This is exacerbated by declining costs and increasing reliance on mobile phones, even in the most resource-poor settings [14]. Communicating information via mobile phones is highly appealing to young people and can positively influence their health outcomes by improving knowledge, reducing sexual risk behavior, and increasing the use of health services [16]. Additionally, mobile phones offer more privacy compared to face-to-face meetings with researchers or health care providers [15], an aspect embraced by AYA. Furthermore, mobile phone solutions may help users overcome barriers to accessibility by soliciting and providing accurate, timely, and engaging information and appropriate care related to highly sensitive topics [16]. Therefore, using a piloted, interactive, digital survey tool, this study adopted a comprehensive psychological, social, and developmental perspective focusing on variables related to AYA’s IPV experiences in Kenya. Our digital survey tool assessed behavioral and psychosocial aspects among AYA between the ages of 15 and 24 years; the prevalence of IPV among AYA in Nairobi was determined and factors associated with IPV risk among AYA were identified.

## Methods

### About the App

The REACH (Reaching, Engaging Adolescents and Young Adults for Care Continuum in Health)-AYA interactive mobile app survey combines existing screening tools, adapted for use jointly with AYA in Kenya through a co-design process. The tools, which were reported in our other published studies, include the WHO, multicountry, gender-based violence study screening tool; the HEEADSSS (Home, Education/employment, Eating, Activities and peer relations, Drugs and alcohol, Sexuality, Suicide/depression, and Safety) assessment instrument [17]; the CRAFFT (Car, Relax, Alone, Forget, Friends, Trouble) screening tool [18]; and the RAST (Risk Assessment Screening Tool) that was adapted by the Kenya Ministry of Health. The app required location access in order to ensure participants resided within Nairobi. Participants did not have to complete the questions all at one time and had the ability to change responses as needed. The survey was structured into 10 modules: (1) social network and support, (2) education, (3) home and family, (4) media and internet use, (5) alcohol and drugs, (6) sexuality, (7) use and perception of health services, (8) mental health, (9) gender and social norms, and (10) religion and spirituality. The app was downloaded from Google Play or the Apple Store and could be used offline only to communicate with the REACH web application once the survey was completed. Data were then synchronized with the hosting server over the mobile network.

### Study Implementation

Prior to study implementation, a team of AYA researchers, health care professionals experienced with AYA, and Nairobi AYA reviewed the data collection instrument and mobile app design. A pilot was conducted to access the mobile app and iterations were performed. We tested the app on 13 potential users selected from our institutional community youth programs from a neighboring county—Kiambu County, due to its proximity and similarity to Nairobi—in order to gain a sense of usability and determine where it may need improvements. Testing assessed three constructs: (1) functionality, (2) time, and (3) adaptability. As part of the iterative process, it was further vetted* *among 33 AYA, aged 15 to 24 years, who provided user feedback to specific questions. Once the technical and business requirements were met, the app was ready for launch and beta testing among a broader group of young people; further information on the pilot test is included in a manuscript that is currently under review.

The study targeted 1061 AYA between the ages of 15 and 24 years. Sample size was calculated for a simple random sample without replacement and adjusted for the clustered nature of the survey and for the finite population correction, yielding a total sample size of 564. Respondent-driven sampling was used for this study population. The theoretical advantage of using respondent-driven sampling among a seldom-heard population is that the dual-incentive system of financial reward in combination with peer pressure could reduce nonresponse bias, since those who would not participate for financial reasons alone may do so as a favor to a friend [19].

Our REACH-AYA Facebook page [20] was used to initiate recruitment; the recruitment process was monitored by our website [21] to ensure that all age groups were represented. Study participants each received 300 Kenyan shillings (US $3) electronically once they completed the survey and an additional 100 Kenyan shillings (US $1) of airtime (ie, phone credit that could be used for calling or browsing) for each friend that was referred and completed the survey, up to a maximum of five friends. All payments and transactions were automated via phone (ie, mobile phone transfer) to minimize human interaction and to promote confidentiality.

Data were captured using Microsoft Excel 2018 with an interface to the app-based survey. As part of the app’s download process, all consenting and enrolled participants were uniquely identified by a participant ID on the central database in Microsoft Excel. Participants within a social network were additionally linked by a network ID.

### Data Analysis

The analysis of data from the formative study included the following components: (1) prevalence of IPV and descriptive analysis of participants’ sociodemographics and (2) descriptive, bivariate, and multivariate analysis of factors contributing to protective and risk factors associated with experiencing IPV.

### Outcome Variable

Intimate violence was classified as a composite variable of physical, sexual, and emotional violence. To determine the presence of physical violence, women were asked whether a current or former partner had ever slapped her or thrown something at her that could hurt her; pushed or shoved her; hit her with a fist or something else that could hurt her; kicked her, dragged her, or beaten her up; choked or burned her on purpose; or threatened her with, or actually used, a gun, knife, or other weapon against her. Sexual violence was defined by the following three behaviors: being physically forced to have sexual intercourse against her will, having sexual intercourse because she was afraid of what her partner might do, or being forced to do something sexual she found degrading or humiliating. Emotional violence included the following: being insulted or made to feel bad about oneself, being humiliated or belittled in front of others, being intimidated or scared on purpose (eg, by a partner yelling and smashing things), or being threatened with harm, directly or indirectly, in the form of a threat to hurt someone the respondent cared about.

### Other Variables

In addition to gender and age, other data collected included sociodemographic data (ie, school status, living arrangements, and economic status), religion and spirituality data, gender norms (ie, beliefs and perceptions of the roles of boys and girls), health activities (ie, sexuality, exercise, and diet), risk behaviors (ie, drugs, alcohol, and sex), psychosocial status (ie, suicidal feelings and depression), and social networks. Coding and statistical analysis were done using Stata 14 (StataCorp). Descriptive statistics were used to summarize participants’ characteristics. Associations between the studied variables and outcome measures were analyzed using bivariate analysis, with all variables that had *P* values of .05 or lower being subjected to multivariable logistic regression. The magnitude of association was measured using adjusted odds ratios (ORs) and 95% CIs. *P* values lower than .05 were considered statistically significant.

### Ethical Approval

Approval for this study was obtained from both the Ethics and Scientific Review Committee of Amref Health Africa in Kenya and the Institutional Review Board of the University of West Florida. All ethical procedures were conducted and maintained throughout the study period.

## Results

### IPV Prevalence

The prevalence of IPV in our population was 22.3% (124/556). A significantly higher proportion of male participants (64/124, 51.6%) had experienced IPV compared to female participants (60/124, 48.4%) (*P*=.01). Age was not included in the analysis tables, as it was not associated with IPV (see Multimedia Appendix 1).

### IPV, Social Network, and Behavioral Risk Factors

#### Bivariate Comparison of Participant Characteristics and Gender

A significantly higher proportion of females (218/320, 68.1%) versus males (308/526, 58.6%) had used the internet to search for health information. There was a significant difference between males (143/318, 45.0%) and females (231/505, 45.7%) who claimed that their friends sometimes let them down. Compared to their female counterparts (212/504, 42.1%), a significantly higher proportion of males (159/313, 50.8%) reported to have never been bullied in school.

#### Bivariate Comparison of Participant Characteristics and IPV Prevalence

Variables that were significantly associated with IPV included respondents doing the following: using the internet to search for information about health issues, reporting that sometimes their friends asked them to do things that they were not very sure about, being criticized by friends, being made angry by friends, often being let down by friends, socializing with a diverse crowd, being bullied at school, having been suspended from school, having had friends or family who used tobacco, and having used illegal drugs to get high (*P*<.05).

### Regression Analysis

Upon unadjusted analysis, male respondents were significantly less likely to experience IPV as compared to females (OR 0.62, 95% CI 0.41-0.92). Respondents who were rarely criticized by their friends were 74% less likely to experience IPV as compared to those who were criticized most of the time (OR 0.26, 95% CI 0.14-0.49). Similarly, the probability of experiencing IPV was reduced by 79% (OR 0.21, 95% CI 0.08-0.56) among respondents who claimed that their friends never let them down as compared to those who were let down most of the time. Respondents who were rarely made angry by friends were significantly less likely to experience IPV as compared to those who were made angry most of the time (OR 0.23, 95% CI 0.13-0.43). Those who socialized with people of the opposite sex were twice as likely to experience IPV relative to those who socialized with a diverse crowd (OR 2.12, 95% CI 1.32-3.40). Similarly, those who were bullied just once were twice as likely to experience IPV relative to those who had never been bullied (OR 2.01, 95% CI 1.17-3.44). The likelihood of experiencing IPV increased by 90% among respondents who had repeated a class in school in the past two years (OR 1.90, 95% CI 1.11-3.23). Respondents living in households that had physical violence or abuse were 3 times more likely to experience IPV relative to those living in homes with no physical violence or abuse (OR 3.70, 95% CI 2.27-6.04).

Respondents whose friends or family used tobacco were twice as likely to experience IPV as compared to those whose friends or family did not (OR 2.55, 95% CI 1.65-3.95). Similarly, the likelihood of experiencing IPV significantly increased by 80% among respondents who had friends or family members who used alcohol (OR 1.80, 95% CI 1.09-2.98). Respectively, during the previous 12 months, respondents who drank a few sips of alcohol and smoked marijuana or hashish were more likely to experience IPV (OR 1.64, 95% CI 1.08-2.49; and OR 1.84, 95% CI 1.17-2.87). The likelihood of experiencing IPV significantly increased by over 2 folds among respondents who had visited a health facility or doctor to receive reproductive health services (OR 2.23, 95% CI 1.40-3.70). The probability of experiencing IPV significantly decreased by 63% among respondents who refused to have sex with someone who was not prepared to use a condom (OR 0.37, 95% CI 0.16-0.85).

Upon adjusted analysis, the likelihood of experiencing IPV significantly increased by over 9 folds among respondents who had been suspended from school as compared to those who considered dropping out (OR 9.73, 95% CI 1.26-75.26). Respondents who resided in homes with physical violence or abuse were 8 times more likely to experience IPV as compared to those that did not (OR 8.90, 95% CI 1.43-55.42).

### Availability of Data

Data used in the analyses for this study are available upon request from the corresponding author.

## Discussion

### Principal Findings

This study assessed behavioral and psychosocial aspects among AYA between the ages of 15 and 24 years as well as the prevalence of IPV among AYA in Nairobi. Consistent with other studies, these findings demonstrate that females were more likely to experience IPV than males [22,23]. In addition, the IPV prevalence of 22.3% among the AYA, 15 to 24 years of age, who participated in the survey is consistent with other global findings among 15-49-year-old women, where IPV prevalence ranged from 15% to 71% in sub-Saharan Africa [24,25]. In relation to social well-being, these findings demonstrate that friendship ties were linked to IPV, thus calling for improved utilization of such ties in understanding IPV among AYA. The interaction between peer influence and adolescent behavior [26,27] was based on homophily theories [28], which state that similarities between AYA and their friends are due to socialization effects. Based on the findings, socialization across genders has different effects on the likelihood of experiencing IPV. Respondents who socialized with people of the opposite sex were twice as likely to experience IPV in contrast to those who socialized with a mixed crowd.

Positive peer influences are linked to protective behaviors among AYA, while negative peer influences are linked to risky behaviors [29]. For example, in the findings, respondents who were satisfied with their social networks and had positive peer interactions (ie, were rarely let down, less criticized, and less angry) were less likely to experience IPV.

IPV is characterized by violence, which is a learned behavior, such that those AYA brought up in violent homes are more likely to perpetuate or suffer from IPV [30-33]. Concurrent with other studies, this study established that respondents who resided in homes with physical violence or abuse were 8 times more likely to experience IPV as compared to those who did not. Children brought up in violent homes are likely to use violence in interpersonal relationships to dominate others, based on the influence of the modeled behavior [34-36]. Additionally, children who see their parents use a weapon are more likely to commit an offense involving a weapon as an adult [37]. Adolescents’ peer relationships are influential and can adversely impact their behaviors [38]. Supportive peer relationships serve as a buffer against violence [39]. The findings from this study established that those who experienced bullying, repeated a class in school, and were suspended from school were more likely to experience IPV compared to their peers who had not gone through such experiences. Programs that seek to utilize schools to prevent IPV among AYA [40] should, therefore, accord special attention to students who have been suspended and/or have undergone bullying.

Our findings demonstrate that participants who used or had social networks that used tobacco, alcohol, or marijuana were more likely to experience IPV compared to those not indulging in such maladaptive behaviors. Deviant peers are linked to a diverse range of delinquent behaviors, including drug use [27,41-43]. Drug and alcohol use are associated with the perpetration of dating- and gender-based violence [44] and physical assault [45]. While a causal relationship between IPV and substance use and abuse cannot be inferred, a positive correlation has been documented [25,46].

Based on the findings, respondents who refused to have sex with someone who was not prepared to use a condom had a significantly reduced likelihood of experiencing IPV. Such findings are encouraging because previous studies have demonstrated that IPV and condom use have an inverse relationship. For example, men who perpetrated violence against their female partners were less likely to engage in consistent condom use [47].

Regarding health information, our findings demonstrate that the majority of the participants were savvy with technology and knew how to find information on the internet related to health issues regarding their bodies, sex, or general issues. The internet supports health-related services among AYA [48], and such health information–seeking behavior may reduce the chances of IPV among AYA; hence, targeted utilization of the internet could be an alternative method for supporting women experiencing IPV [49,50]. Nevertheless, the internet has a lot of inaccurate information, so AYA should be made aware of legitimate websites and how to screen out inaccurate information.

The likelihood of experiencing IPV significantly increased by over 2 folds among respondents who had visited a health facility or doctor to receive reproductive health services. Given that health systems provide a suitable entry point for the advancement of well-being for adolescents [51], these findings affirm the need to explore the integration of appropriate IPV-preventive strategies within youth-friendly clinics.

### Novelty of the Results From This Study

In the adolescent space, friendship ties play a significant role since they are the most salient networks through which behavioral and normative influences are shared. This study, therefore, clearly highlights the significance of adolescent friendship ties as avenues to understand the context of IPV among AYA. These networks can be leveraged to address occurrences of IPV and to examine the risk of IPV among adolescents.

### Limitations

This study utilized a cross-sectional sample that may limit the likelihood of generalizing findings to the general population of AYA. In addition, the app was only accessible to people who could download it on their mobile phones or other technological apparatus. Thus, information from AYA who are not technologically savvy or from those who experienced technological difficulties was not collected. In future, such youth can serve as the control sample. In addition, the study sample was only from one part of Kenya (ie, Nairobi), which limits comparison with other AYA in other cities. Nevertheless, this study served as a pilot that can be enhanced and rolled out to other cities in Kenya and Africa.

### Conclusions

Maladaptive friendship ties, violence at home, health information–seeking behaviors, and alcohol and drug use have significant roles in the experience of IPV and can be utilized to identify the risk and protective factors associated with IPV among AYA. IPV-prevention strategies in this vulnerable population should be contextualized to meet targeted needs. Efforts toward integrating IPV prevention in youth-friendly clinics is critical. In addition, adequate funding and policies that support such efforts are necessary.

## Appendix

Multimedia Appendix 1 Participant characteristics and risk factors associated with intimate partner violence (IPV).

## Abbreviations

AYA: adolescents and young adults
CODESRIA: Council for the Development of Social Science Research in Africa
CRAFFT: Car, Relax, Alone, Forget, Friends, Trouble
HEEADSSS: Home, Education/employment, Eating, Activities and peer relations, Drugs and alcohol, Sexuality, Suicide/depression, and Safety
HISTP: HIV Intervention Science Training Program
IPV: intimate partner violence
OR: odds ratio
RAST: Risk Assessment Screening Tool
REACH: Reaching, Engaging Adolescents and Young Adults for Care Continuum in Health
WHO: World Health Organization
