# Supplementary material for: Using Friendship Ties to Understand the Prevalence of, and Factors Associated With, Intimate Partner Violence Among Adolescents and Young Adults in Kenya: Cross-Sectional, Respondent-Driven Survey Study
Source: Interact J Med Res. 2020 Dec 31;9(4):e19023. doi: 10.2196/19023 (PMC7808892; doi:10.2196/19023)
Supplement: Multimedia Appendix 1 [file ijmr_v9i4e19023_app1.docx]

**Table S1. Participant Characteristics by Sex and Intimate Partner Violence (IPV)**

|  | **Total % (n)** | **Sex % (n)** | | |  | **Intimate Partner Violence % (n)** | | |
| --- | --- | --- | --- | --- | --- | --- | --- | --- |
| **Characteristic** |  | **Male** | **Female** | **p value** |  | **Experienced IPV** | **No IPV** | **p value** |
| Total | 887 |  |  |  |  | 22.3 (124) | 77.7 (432) |  |
| **Gender** |  |  |  |  |  |  |  | **0.018** |
| Female | 39.1 (347) |  |  |  |  | 48.4 (60) | 36.6 (158) |  |
| Male | 60.9 (540) |  |  |  |  | 51.6 (64) | 63.4 (274) |  |
| ***Social Network*** |  |  |  |  |  |  |  |  |
| **Where do look for information about health issues about your body or sex or general issues** (n =829) |  |  |  | **0.029** |  |  |  | **<0.001** |
| My friends on social media | 8.4 (70) | 8.9 (47) | 7.2 (23) |  |  | 8.9 (11) | 7.1 (30) |  |
| My parents | 13 (108) | 12.7 (67) | 12.8 (41) |  |  | 24.4 (30) | 8.5 (36) |  |
| My relatives | 9.3 (77) | 9.1 (48) | 9.1 (29) |  |  | 6.5 (8) | 9.2 (39) |  |
| My siblings | 5.8 (48) | 7.4 (39) | 2.8 (9) |  |  | 7.3 (9) | 4.2 (18) |  |
| The internet – Google | 63.4 (526) | 58.4 (308) | 68.1 (218) |  |  | 52.8 (65) | 71.1 (302) |  |
| **How often do your friends ask you to do something you are not very sure about or that you don’t feel like doing?** (n =822) |  |  |  | 0.092 |  |  |  | **0.011** |
| Most Times | 14.2 (117) | 11.3 (36) | 16.1 (81) |  |  | 19.7 (24) | 13.7 (58) |  |
| Never | 6.7 (55) | 7.2 (23) | 6.4 (32) |  |  | 4.1 (5) | 4.5 (19) |  |
| Rarely | 33.5 (275) | 31.3 (100) | 34.8 (175) |  |  | 20.5 (25) | 35.9 (152) |  |
| Sometimes | 45.6 (375) | 50.2 (160) | 42.7 (215) |  |  | 55.7 (68) | 45.9 (194) |  |
| **How often do your friends criticize you?** (n =829) |  |  |  | 0.781 |  |  |  | **<0.001** |
| Most Times | 14.2 (118) | 15.3 (49) | 13.6 (69) |  |  | 25.2 (31) | 12.4 (52) |  |
| Never | 9.3 (77) | 9.7 (31) | 9.0 (46) |  |  | 5.7 (7) | 9.3 (39) |  |
| Rarely | 37.3 (309) | 35.3 (113) | 38.5 (196) |  |  | 21.1 (26) | 39.2 (165) |  |
| Sometimes | 39.2 (325) | 39.7 (127) | 38.9 (198) |  |  | 48 (59) | 39.2 (165) |  |

**Table S1. Participant Characteristics by Sex and Intimate Partner Violence (IPV)**

|  | **Total % (n)** | | **Sex % (n)** | | | | | |  | | **Intimate Partner Violence % (n)** | | | | | |
| --- | --- | --- | --- | --- | --- | --- | --- | --- | --- | --- | --- | --- | --- | --- | --- | --- |
| **Characteristic** |  |  | **Male** | | **Female** | | **p value** | |  | | **Experienced IPV** | | **No IPV** | | **p value** | |
| **How often do they let you down when you are counting on them?** (n =823) |  | |  | |  | | **0.010** | |  | |  | |  | | **<0.001** | |
| Most Times | 14.7 (121) | | 19.2 (61) | | 11.9 (60) | |  | |  | | 30.9 (38) | | 10.4 (44) | |  | |
| Never | 8.5 (70) | | 6.0 (19) | | 10.1 (51) | |  | |  | | 4.9 (6) | | 7.8 (33) | |  | |
| Rarely | 31.3 (258) | | 29.9 (95) | | 32.3 (163) | |  | |  | | 26.0 (32) | | 29.1 (123) | |  | |
| Sometimes | 45.4 (374) | | 45.0 (143) | | 45.7 (231) | |  | |  | | 38.2 (47) | | 52.6 (222) | |  | |
| **How often do your friends get you angry?** (n =824) |  | |  | |  | | 0.301 | |  | |  | |  | | **<0.001** | |
| Most Times | 9.6 (79) | | 11.3 (36) | | 8.5 (43) | |  | |  | | 24.6 (30) | | 8.1 (34) | |  | |
| Never | 5.0 (41) | | 3.8 (12) | | 5.8 (29) | |  | |  | | 2.5 (3) | | 2.8 (12) | |  | |
| Rarely | 39.2 (323) | | 37.5 (120) | | 40.3 (203) | |  | |  | | 27.9 (34) | | 39.1 (165) | |  | |
| Sometimes | 46.2 (381) | | 47.5 (152) | | 45.4 (229) | |  | |  | | 45.1 (55) | | 50.0 (211) | |  | |
| ***Education*** |  | |  | |  | |  | |  | |  | |  | |  | |
| **You socialize with?** (n =820) |  | |  | |  | | 0.684 | |  | |  | |  | | **0.004** | |
| A mixed crowd | 74.1 (608) | | 74.3 (234) | | 74.1 (374) | |  | |  | | 65.8 (79) | | 76.9 (326) | |  | |
| People of the opposite gender | 17.9 (147) | | 18.7 (59) | | 17.4 (88) | |  | |  | | 30.0 (36) | | 16.5 (70) | |  | |
| People of the same gender | 7.9 (65) | | 7.0 (22) | | 8.5 (43) | |  | |  | | 4.2 (5) | | 6.6 (28) | |  | |
| **Ever bullied at school?** (n =817) |  | |  | |  | | **0.020** | |  | |  | |  | | **0.026** | |
| Never | 45.4 (371) | | 50.8 (159) | | 42.1 (212) | |  | |  | | 32.2 (39) | | 47.8 (202) | |  | |
| Once | 20.1 (164) | | 18.5 (58) | | 21.0 (106) | |  | |  | | 25.6 (31) | | 18.9 (80) | |  | |
| Several times | 8.2 (67) | | 5.1 (16) | | 10.1 (51) | |  | |  | | 9.1 (11) | | 7.3 (31) | |  | |
| Sometimes | 26.3 (215) | | 25.6 (80) | | 26.8 (135) | |  | |  | | 33.1 (40) | | 26.0 (110) | |  | |
| **In the last 2 years, have you had to repeat a class?** (n =769) | |  | |  |  | 0.910 | |  | |  | |  | | **0.017** | |  |
| No | | 85.8 (660) | | 86.0 (252) | 85.7 (408) |  | |  | | 78.3 (90) | | 87.2 (348) | |  | |  |
| Yes | | 14.2 (109) | | 14.0 (41) | 14.3 (68) |  | |  | | 21.7 (25) | | 12.8 (51) | |  | |  |
| **Have you ever** (n =811)**:** | |  | |  |  | 0.704 | |  | |  | |  | | **0.030** | |  |
| Considered dropping out | | 12.1 (98) | | 11.9 (37) | 12.2 (61) |  | |  | | 20.7 (25) | | 12.1 (51) | |  | |  |
| Been Expelled | | 3.8 (31) | | 3.8 (12) | 3.8 (19) |  | |  | | 2.5 (3) | | 2.9 (12) | |  | |  |
| None of the above | | 67.8 (550) | | 69.9 (218) | 66.5 (332) |  | |  | | 54.5 (66) | | 67.9 (286) | |  | |  |
| Suspended | | 16.3 (132) | | 14.4 (45) | 17.4 (87) |  | |  | | 22.3 (27) | | 17.1 (72) | |  | |  |

**Table S1. Participant Characteristics by Sex and Intimate Partner Violence (IPV)**

|  | **Total % (n)** | **Sex % (n)** | | |  | **Intimate Partner Violence % (n)** | | |
| --- | --- | --- | --- | --- | --- | --- | --- | --- |
| **Characteristic** |  | **Male** | **Female** | **p value** |  | **Experienced IPV** | **No IPV** | **p value** |
| ***Home and Family*** |  |  |  |  |  |  |  |  |
| **Do you get along with your family?** (n =807) |  |  |  | 0.841 |  |  |  | 0.105 |
| Mostly yes | 79.3 (640) | 78.8 (242) | 79.6 (398) |  |  | 74.4 (90) | 82.9 (350) |  |
| Not at all | 7.2 (58) | 6.8 (21) | 7.4 (37) |  |  | 7.4 (9) | 4.7 (20) |  |
| Sometimes | 13.5 (109) | 14.3 (44) | 13 (65) |  |  | 18.2 (22) | 12.3 (52) |  |
| **Is there any physical violence/abuse at home?** (n =801) |  |  |  | 0.388 |  |  |  | **<0.001** |
| No | 83.5 (669) | 82.1 (252) | 84.4 (417) |  |  | 67.8 (80) | 88.6 (374) |  |
| Yes | 16.5 (132) | 17.9 (55) | 15.6 (77) |  |  | 32.2 (38) | 11.4 (48) |  |
| **Have you ever run away from home?** (n =804) |  |  |  | 0.54 |  |  |  | **<0.001** |
| No | 79.5 (639) | 78.4 (239) | 80.2 (400) |  |  | 66.1 (80) | 82.5 (349) |  |
| Yes | 20.5 (165) | 21.6 (66) | 19.8 (99) |  |  | 33.9 (41) | 17.5 (74) |  |
| ***Alcohol and Drugs*** |  |  |  |  |  |  |  |  |
| **Do any of your friends or family use Tobacco?** (n =733) |  |  |  | **0.03** |  |  |  | **<0.001** |
| No | 58 (425) | 62.9 (180) | 54.8 (245) |  |  | 36.4 (40) | 59.3 (235) |  |
| Yes | 42 (308) | 37.1 (106) | 45.2 (202) |  |  | 63.6 (70) | 40.7 (161) |  |
| **Do any of your friends or family use Alcohol** (n =742) |  |  |  | 0.837 |  |  |  | **0.02** |
| No | 32.6 (242) | 32.2 (92) | 32.9 (150) |  |  | 19.3 (23) | 30.1 (123) |  |
| Yes | 67.4 (500) | 67.8 (194) | 67.1 (306) |  |  | 80.7 (96) | 69.9 (285) |  |
| **During the past 12 months, did you drink any alcohol (more than a few sips)?** (n =748) |  |  |  | 0.303 |  |  |  | **0.02** |
| No | 53.3 (399) | 55.7 (161) | 51.9 (238) |  |  | 37.0 (44) | 49.0 (202) |  |
| Yes | 46.7 (349) | 44.3 (128) | 48.1 (221) |  |  | 63.0 (75) | 51.0 (210) |  |

**Table S1. Participant Characteristics by Sex and Intimate Partner Violence (IPV)**

|  | **Total % (n)** | **Sex % (n)** | | |  | **Intimate Partner Violence % (n)** | | |
| --- | --- | --- | --- | --- | --- | --- | --- | --- |
| **Characteristic** |  | **Male** | **Female** | **p value** |  | **Experienced IPV** | **No IPV** | **p value** |
| **During the past 12 months, did you Smoke any marijuana or hashish?** (n =731) |  |  |  | 0.129 |  |  |  | **0.007** |
| No | 76.9 (562) | 79.9 (226) | 75.0 (336) |  |  | 63.1 (70) | 75.8 (304) |  |
| Yes | 23.1 (169) | 20.1 (57) | 25.0 (112) |  |  | 36.9 (41) | 24.2 (97) |  |
| **During the past 12 months, did you Use anything else to get high?** (n =728) |  |  |  | 0.619 |  |  |  | **<0.001** |
| No | 83.7 (609) | 84.5 (240) | 83.1 (369) |  |  | 64.7 (77) | 88.8 (348) |  |
| Yes | 16.3 (119) | 15.5 (44) | 16.9 (75) |  |  | 35.3 (42) | 11.2 (44) |  |
| **Have you ever ridden in a car driven by someone (including yourself) who was “high” or had been using alcohol or drugs?”** (n =732) |  |  |  | 0.345 |  |  |  | **0.002** |
| No | 58.2 (426) | 60.4 (172) | 56.8 (254) |  |  | 43.1 (50) | 59.3 (237) |  |
| Yes | 41.8 (306) | 39.6 (113) | 43.2 (193) |  |  | 56.9 (66) | 40.8 (163) |  |
| **Do you ever use alcohol or drugs to relax, feel better about yourself, or fit in?** (n =720) |  |  |  | **0.06** |  |  |  | **<0.001** |
| No | 69.9 (503) | 73.9 (209) | 67.3 (294) |  |  | 49.2 (58) | 71.5 (278) |  |
| Yes | 30.1 (217) | 26.1 (74) | 32.7 (143) |  |  | 50.8 (60) | 28.5 (111) |  |
| **Do you ever use alcohol or drugs while you are alone?** (n =721) |  |  |  | 0.175 |  |  |  | **<0.001** |
| No | 73.1 (527) | 75.9 (214) | 71.3 (313) |  |  | 51.7 (61) | 75.8 (297) |  |
| Yes | 26.9 (194) | 24.1 (68) | 28.7 (126) |  |  | 48.3 (57) | 24.2 (95) |  |

**Table S1. Participant Characteristics by Sex and Intimate Partner Violence (IPV)**

|  | **Total % (n)** | **Sex % (n)** | | |  | **Intimate Partner Violence % (n)** | | |
| --- | --- | --- | --- | --- | --- | --- | --- | --- |
| **Characteristic** |  | **Male** | **Female** | **p value** |  | **Experienced IPV** | **No IPV** | **p value** |
| **I am confident that I can insist on condom use every time I have sex** (n =630) |  |  |  | 0.821 |  |  |  | 0.130 |
| Disagree | 13.3 (84) | 13.4 (31) | 13.3 (53) |  |  | 16.0 (16) | 13.6 (46) |  |
| Agree | 67.1 (423) | 65.8 (152) | 67.9 (271) |  |  | 62.0 (62) | 71.9 (243) |  |
| Don't know/not sure | 19.5 (123) | 20.8 (48) | 18.8 (75) |  |  | 22.0 (22) | 14.5 (49) |  |
| **It is mainly the woman's responsibility to ensure that contraception is used regularly** (n =632) |  |  |  | 0.079 |  |  |  | 0.514 |
| Don't know/not sure | 18.5 (117) | 14.0 (32) | 21.0 (85) |  |  | 17.6 (18) | 14.8 (50) |  |
| Agree | 31.2 (197) | 34.2 (78) | 29.5 (119) |  |  | 34.3 (35) | 30.8 (104) |  |
| Disagree | 50.3 (318) | 51.8 (118) | 49.5 (200) |  |  | 48.1 (49) | 54.4 (184) |  |
| **I would refuse to have sex with someone who is not prepared to use a condom** (n =611) |  |  |  | 0.959 |  |  |  | **0.007** |
| Don't know/not sure | 19.3 (118) | 19.9 (45) | 19.0 (73) |  |  | 27.3 (27) | 13.9 (46) |  |
| Agree | 68.6 (419) | 68.1 (154) | 68.8 (265) |  |  | 62.6 (62) | 72.2 (240) |  |
| Disagree | 12.1 (74) | 11.9 (27) | 12.2 (47) |  |  | 10.1 (10) | 13.9 (46) |  |

**Table S2. Risk Factors Associated with IPV**

|  |  | **Unadjusted OR** | | |  | **Adjusted OR** | | |
| --- | --- | --- | --- | --- | --- | --- | --- | --- |
| **Characteristic** | **Prevalence % (n)** | **uOR** | **95% CI** | **p-value** |  | **aOR** | **95%CI** | **p-value** |
| **Gender** |  |  |  |  |  |  |  |  |
| Female | 48.4 (60) | 1 | - |  |  |  |  |  |
| Male | 51.6 (64) | 0.62 | 0.41 - 0.92 | **0.0181** |  | 0.46 | 0.14 - 1.54 | 0.21 |
| ***Social Network*** |  |  |  |  |  |  |  |  |
| **Where do look for information about health issues about your body or sex or general issues** |  |  |  |  |  |  |  |  |
| My friends on social media | 8.9 (11) | 1 | - |  |  | 1 |  |  |
| My parents | 24.4 (30) | 2.27 | 0.98 - 5.28 | 0.0565 |  | 6.77 | 0.42 - 108.33 | 0.18 |
| My relatives | 6.5 (8) | 0.56 | 0.20 - 1.56 | 0.2679 |  | 0.31 | 0.01 - 6.53 | 0.45 |
| My siblings | 7.3 (9) | 1.36 | 0.47 - 3.92 | 0.5653 |  | 2.53 | 0.12 - 52.03 | 0.55 |
| The internet – Google | 52.8 (65) | 0.59 | 0.28 - 1.23 | 0.1588 |  | 1.18 | 0.15 - 9.5 | 0.88 |
| **How often do your friends ask you to do something you are not very sure about or that you don’t feel like doing?** |  |  |  |  |  |  |  |  |
| Most Times | 19.7 (24) | 1 | - |  |  | 1 | - |  |
| Never | 4.1 (5) | 0.64 | 0.21 - 1.90 | 0.4174 |  | 0.74 | 0.01 - 38.05 | 0.88 |
| Rarely | 20.5 (25) | 0.4 | 0.21 - 0.75 | 0.0045 |  | 0.17 | 0.03 - 1.08 | 0.06 |
| Sometimes | 55.7 (68) | 0.85 | 0.49 - 1.47 | 0.5543 |  | 0.27 | 0.05 - 1.57 | 0.14 |
| **How often do your friends criticize you?** |  |  |  |  |  |  |  |  |
| Most Times | 25.2 (31) | 1 | - |  |  | 1 |  |  |
| Never | 5.7 (7) | 0.3 | 0.12 - 0.75 | **0.0105** |  | 0.08 | 0 - 4.43 | 0.22 |
| Rarely | 21.1 (26) | 0.26 | 0.14 - 0.49 | **<0.001** |  | 0.45 | 0.07 - 2.8 | 0.39 |
| Sometimes | 48 (59) | 0.6 | 0.35 - 1.02 | 0.0611 |  | 0.77 | 0.17 - 3.5 | 0.74 |

**Table S2. Risk Factors Associated with IPV**

|  |  | **Unadjusted OR** | | |  | **Adjusted OR** | | |
| --- | --- | --- | --- | --- | --- | --- | --- | --- |
| **Characteristic** | **Prevalence % (n)** | **uOR** | **95% CI** | **p-value** |  | **aOR** | **95%CI** | **p-value** |
| **How often do they let you down?**  Never | 4.9 (6) | 0.21 | 0.08 – 0.56 | **0.0017** |  | - |  |  |
| Rarely | 26 (32) | 0.3 | 0.17 – 0.54 | **<0.001** |  | 0.08 | 0.01 – 0.5 | **0.01** |
| Sometimes | 38.2 (47) | 0.25 | 0.14 – 0.42 | **<0.001** |  | 0.11 | 0.02 – 0.52 | **0.01** |
| **How often do your friends get you angry?** |  |  |  |  |  |  |  |  |
| Most Times | 24.6 (30) | 1 | - |  |  | 1 |  |  |
| Never | 2.5 (3) | 0.28 | 0.07 – 1.10 | 0.0685 |  | - |  |  |
| Rarely | 27.9 (34) | 0.23 | 0.13 – 0.43 | **<0.001** |  | 1.05 | 0.15 – 7.15 | 0.96 |
| Sometimes | 45.1 (55) | 0.3 | 0.17 – 0.52 | **<0.001** |  | 0.94 | 0.16 – 5.65 | 0.95 |
| **Whom Do you socialize with?** |  |  |  |  |  |  |  |  |
| A mixed crowd | 65.8 (79) | 1 | - |  |  |  |  |  |
| People of the opposite gender | 30 (36) | 2.12 | 1.32 – 3.40 | **0.0017** |  | 0.56 | 0.11 – 2.91 | 0.49 |
| People of the same gender | 4.2 (5) | 0.74 | 0.28 – 1.97 | 0.5426 |  | 0.21 | 0.01 – 5.02 | 0.34 |
| **Have you been bullied at school?** |  |  |  |  |  |  |  |  |
| Never | 32.2 (39) | 1 | - |  |  | 1 |  |  |
| Once | 25.6 (31) | 2.01 | 1.17 – 3.44 | **0.0112** |  | 2.94 | 0.69 – 12.49 | 0.14 |
| Several times | 9.1 (11) | 1.84 | 0.85 – 3.96 | 0.1206 |  | 0.34 | 0.03 – 3.46 | 0.36 |
| Sometimes | 33.1 (40) | 1.88 | 1.14 – 3.10 | **0.0128** |  | 1.25 | 0.29 - 5.34 | 0.76 |
| **In the last 2 years, have you had to repeat a class?** |  |  |  |  |  |  |  |  |
| No | 78.3 (90) | 1 | - |  |  | 1 |  |  |
| Yes | 21.7 (25) | 1.9 | 1.11 - 3.23 | **0.018** |  | 1.06 | 0.16 - 7.11 | 0.95 |
| **Have you missed school in the last 12 months?** |  |  |  |  |  |  |  |  |
| No | 54.7 (64) | 1 | - |  |  | 1 |  |  |
| Yes | 45.3 (53) | 1.42 | 0.93 - 2.15 | 0.101 |  | 0.67 | 0.2 - 2.22 | 0.51 |
| **Have you ever been:** |  |  |  |  |  |  |  |  |
| Considered dropping out | 20.7 (25) | 1 | - |  |  | 1 |  |  |
| Expelled | 2.5 (3) | 0.51 | 0.13 - 1.97 | 0.329 |  | - |  |  |
| None of the above | 54.5 (66) | 0.47 | 0.27 - 0.81 | 0.007 |  | 3.03 | 0.43 - 21.33 | 0.27 |
| Suspended | 22.3 (27) | 0.76 | 0.40 - 1.47 | 0.420 |  | 9.73 | 1.26 - 25.26 | **0.03** |

**Table S2. Risk Factors Associated with IPV**

|  |  | | | | **Unadjusted OR** | | | | | | | | |  | | | **Adjusted OR** | | | | | | | | |
| --- | --- | --- | --- | --- | --- | --- | --- | --- | --- | --- | --- | --- | --- | --- | --- | --- | --- | --- | --- | --- | --- | --- | --- | --- | --- |
| **Characteristic** |  | | | | **uOR** | | | **95% CI** | | | **p-value** | | |  | | | **aOR** | | | **95%CI** | | | **p-value** | | |
| ***Home and Family*** |  | |  | | |  | | |  | | |  | | |  | | |  | | |  | | |  |  |
| **Do you get along with your family?** |  | |  | | |  | | |  | | |  | | |  | | |  | | |  | | |  |  |
| Mostly yes | 74.4 (90) | | 1 | | | - | | |  | | |  | | | 1 | | |  | | |  | | |  |  |
| Not at all | 7.4 (9) | | 1.75 | | | 0.77 - 3.97 | | | 0.181 | | |  | | | 5.61 | | | 0.34 - 92.85 | | | 0.23 | | |  |  |
| Sometimes | 18.2 (22) | | 1.65 | | | 0.95 - 2.85 | | | 0.075 | | |  | | | 0.96 | | | 0.16 - 5.76 | | | 0.97 | | |  |  |
| **Is there any physical violence/abuse at home?** |  | |  | | |  | | |  | | |  | | |  | | |  | | |  | | |  |  |
| No | 67.8 (80) | | 1 | | | - | | |  | | |  | | |  | | |  | | |  | | |  |  |
| Yes | 32.2 (38) | | 3.7 | | | 2.27 - 6.04 | | | **<0.001** | | |  | | | 8.90 | | | 1.43 - 9.42 | | | **0.02** | | |  |  |
| **Have you ever ridden in a car driven by someone (including yourself) who was “high” or had been using alcohol or drugs?"** | |  | |  | | |  | | |  | | |  | | |  | | |  | | |  | | |  |
| No | | 43.1 (50) | | 1 | | | - | | |  | | |  | | | 1 | | |  | | |  | | |  |
| Yes | | 56.9 (66) | | 1.92 | | | 1.26 - 2.92 | | | **0.002** | | |  | | | 2.47 | | | 0.71 - 8.61 | | | 0.16 | | |  |
| **Do you ever use alcohol or drugs to relax, feel better about yourself, or fit in?** | |  | |  | | |  | | |  | | |  | | |  | | |  | | |  | | |  |
| No | | 49.2 (58) | | 1 | | | - | | |  | | |  | | | 1 | | |  | | |  | | |  |
| Yes | | 50.8 (60) | | 2.59 | | | 1.70 - 3.95 | | | **<0.001** | | |  | | | 1.38 | | | 1.09 - 1.65 | | | **0.02** | | |  |
| **Do you ever use alcohol or drugs while you are alone?** | |  | |  | | |  | | |  | | |  | | |  | | |  | | |  | | |  |
| No | |  | | 1 | | | - | | |  | | |  | | | 1 | | |  | | |  | | |  |
| Yes | |  | | 2.92 | | | 1.90 - 4.48 | | | **<0.001** | | |  | | | 2.76 | | | 0.63 - 12.02 | | | 0.18 | | |  |
| **Do you ever forget things you did while using alcohol or drugs?** | |  | |  | | |  | | |  | | |  | | |  | | |  | | |  | | |  |
| No | | 52 (53) | | 1 | | | - | | |  | | |  | | | 1 | | |  | | |  | | |  |
| Yes | | 48 (49) | | 2.16 | | | 1.37 - 3.40 | | | **<0.001** | | |  | | | 0.85 | | | 0.19 - 3.81 | | | 0.83 | | |  |
| **Do your family or friends ever tell you that you should cut down on your drinking or drug use?** | |  | |  | | |  | | |  | | |  | | |  | | |  | | |  | | |  |
| No | | 56.7 (55) | | 1 | | | - | | |  | | |  | | | 1 | | |  | | |  | | |  |
| Yes | | 43.3 (42) | | 2.02 | | | 1.25 - 3.26 | | | **0.0039** | | |  | | | 2.46 | | | 1.6 - 10.02 | | | 0.13 | | |  |

**Table S2. Risk Factors Associated with IPV**

|  |  | **Unadjusted OR** | | |  | **Adjusted OR** | | |
| --- | --- | --- | --- | --- | --- | --- | --- | --- |
| **Characteristic** |  | **uOR** | **95% CI** | **p-value** |  | **aOR** | **95%CI** | **p-value** |
| **Have you ever gotten into trouble while you were using alcohol or drugs?** |  |  |  |  |  |  |  |  |
| No | 44.1 (41) | 1 | - |  |  | 1 |  |  |
| Yes | 55.9 (52) | 2.92 | 1.81 - 4.72 | **<0.001** |  | 3.65 | 0.84 - 15.78 | 0.08 |
| **Have you ever visited a health facility or doctor or pharmacy/chemist of any kind to receive services or information on contraception, pregnancy, abortion or sexually transmitted diseases?** |  |  |  |  |  |  |  |  |
| No | 21.5 (25) | 1 | - |  |  | 1 |  |  |
| Yes | 78.5 (91) | 2.28 | 1.40 - 3.70 | **<0.001** |  | **2.31** | **1.65 - 8.20** | **0.02** |
| **It is sometimes ok for a boy to hit his girlfriend** |  |  |  |  |  |  |  |  |
| Disagree | 65.1 (67) | 1 | - |  |  | 1 |  |  |
| Don't know/not sure | 19.4 (20) | 2.45 | 1.32 - 4.54 | **0.004** |  | 1.59 | 1.06 - 5.52 | 0.045 |
| Agree | 15.5 (16) | 1.62 | 1.85 - 3.06 | **0.039** |  | 2.39 | 1.42 - 13.47 | **0.024** |
| **I would refuse to have sex with someone who is not prepared to use a condom** |  |  |  |  |  |  |  |  |
| Don't know/not sure | 27.3 (27) | 1 | - |  |  | 1 |  |  |
| Agree | 62.6 (62) | 0.44 | 0.25 - 0.76 | **0.003** |  | 0.96 | 0.22 - 4.2 | 0.953 |
| Disagree | 10.1 (10) | 1.37 | 1.16 - 1.85 | **0.019** |  | 1.23 | 1.02 - 2.27 | **0.007** |

180
